# Robotic Ureteral Reconstruction in the Pediatric Population

**DOI:** 10.3389/fped.2019.00085

**Published:** 2019-03-22

**Authors:** Aylin N. Bilgutay, Andrew J. Kirsch

**Affiliations:** ^1^Department of Pediatric Urology, Children's Healthcare of Atlanta, Atlanta, GA, United States; ^2^Department of Urology, Emory University School of Medicine, Atlanta, GA, United States

**Keywords:** robotic surgery, pediatric urology, pyeloplasty, ureteroureterostomy, vesicoureteral reflux, ureteropelvic junction obstruction, ureterovesical junction obstruction, megaureter

## Abstract

Robot-assisted laparoscopic (RAL) surgery is a safe, minimally invasive technique that has become more widely used in pediatric urology over recent decades. With several advantages over standard laparoscopy, robotic surgery is particularly well-suited to reconstructive surgery involving delicate structures like the ureter. A robotic approach provides excellent access to and visualization of the ureter at all levels. Common applications include upper ureteral reconstruction (e.g., pyeloplasty, ureteropelvic junction polypectomy, ureterocalicostomy, and high uretero-ureterostomy in duplex systems), mid-ureteral reconstruction (e.g., mid uretero-ureterostomy for stricture or polyp), and lower ureteral reconstruction (e.g., ureteral reimplantation and lower ureter-ureterostomy in duplex systems). Herein, we describe each of these robotic procedures in detail.

## Introduction

Many considerations are involved in choosing surgical approach. Compared to open surgery, roboticsoffer several advantages including smaller incisions and more rapid convalescence. Robot-assisted laparoscopic (RAL) surgery may, however, be difficult or even impossible in very small patients, in whom pure laparoscopic intervention may be preferred. Pure laparoscopy allows for even smaller incisions than robotic surgery, with ports as small as 2–3 mm available. Another disadvantage of robotic surgery is increased cost compared to pure laparoscopic or open approaches. Benefits of robotic surgery include wristed movements and magnified vision, making it the ideal approach for delicate reconstructive procedures. Robotics continue to enjoy expanding applications and growing popularity among urologists and patient families alike.

## Upper Ureteral Reconstruction

### Pyeloplasty

Pyeloplasty for ureteropelvic junction (UPJ) obstruction is the most common robotic surgery in pediatric urology (1). RAL retroperitoneoscopic pyeloplasty has been described in children (2); however, the transabdominal approach is more frequently utilized, providing a larger working space that facilitates dissection and anastomosis. Transabdominal robotic approach may involve transmesenteric UPJ exposure for left-sided cases to decrease operative time, as previously described for traditional laparoscopic pyeloplasty (3). However, reflecting the colon is usually rapid, and one should not risk limited exposure for potential time savings, particularly in complex cases.

Prior to positioning for the robotic portion of the case, we prefer starting with cystoscopy and retrograde pyelogram to delineate anatomy unless adequately assessed preoperatively with magnetic resonance urogram. A ureteral stent may be placed retrograde if desired. We prefer placing a ureteral stent antegrade during the robotic portion of the case.

Typical patient positioning for transabdominal robotic pyeloplasty is the modified flank/lateral decubitus position with affected side elevated ~45° over a roll, contralateral arm extended on an arm board, and ipsilateral arm straight against the patient's ipsilateral side or extended parallel to the contralateral arm using an elevated armrest or pillows. Alternatively, the patient may be positioned supine with table rotation to elevate the pathologic side (1). One must ensure that all pressure points are adequately padded and the patient is appropriately secured to the table.

The patient is flattened for port placement. The camera port is placed first, usually at the umbilicus, using either open Hasson or Veress needle technique. Some surgeons recommend against the use of Veress needle in children (4). However, we believe that this technique can be applied safely in pediatric cases and have successfully used it for several years at our high volume robotic institution with no complications. For the Si, we use the 8.5 mm robotic camera port. A 10 or 12 mm port (e.g., the Autosuture^®^ balloon trocar) may also be used as the Si robot camera port (5). For the Xi, the camera and working ports are identical, allowing placement of the camera through any port. Robotic working ports are then placed under direct vision. For the Si, 8 and 5 mm robotic ports and instruments are available, while only 8 mm ports/instruments are available for the Xi. We prefer 8 mm robotic ports even with the Si because of the greater variety of instrumentation available. Another advantage of the 8 mm instruments is a shorter intracorporeal length of the wristed segment, with decreased required intracorporeal working distance (5).

**Figure 1:**
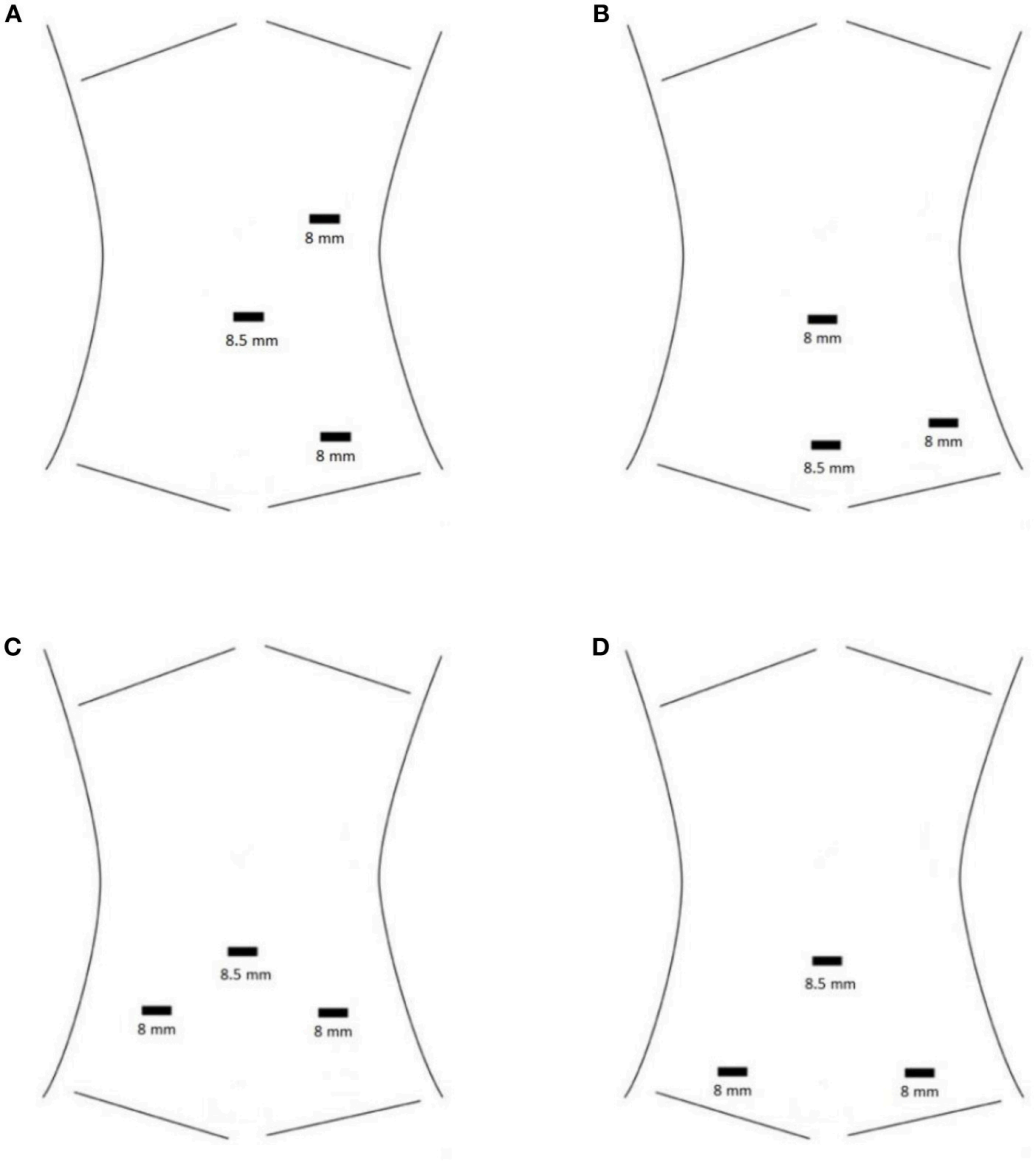
**(A)** Standard port placement for RAL left pyeloplasty with the Si robot. The camera port is at the umbilicus. **(B)** HIdES port placement for RAL left pyeloplasty with the Si robot. The camera port is the inferior-most port. The camera port and one working port are hidden at or below the level of a Pfannenstiel incision, while the other working port is hidden in the umbilicus. **(C)** Standard port placement for RAL ureteral reimplantation with the Si robot. The camera port is at the umbilicus. **(D)** HIdES port placement for RAL ureteral reimplantation with the Si robot. The camera port is at the umbilicus. Skin incisions for the working ports are lower than in the standard port placement (at or below the level of a Pfannenstiel incision). Fascial entry sites for the working ports may be placed higher than the skin incisions in order to increase working space within the pelvis. This is achieved by applying cephalad traction during port placement.

For the Si, ideal port placement results in a triangular working field. One working port is placed cephalad to the camera port in the midline or midclavicular line, and the other is placed inferiorly at an ~30° angle rotated from midline toward the kidney of interest (Figure 1A). Ports are ideally spaced ~1 hand's breadth apart, but this may be impossible in smaller children and infants. All ports are instead placed in the midline to maximize the limited working space in infants, as close as 3 cm if necessary (5).

Optimal port placement for the Xi robot is in a line rather than triangulated. A third robotic port and/or assistant port(s) may be placed if desired. We usually do not find additional ports necessary. With our typical three-port setup, a robotic instrument must be removed to allow the assistant to suction or pass sutures. This positioning and port placement may be used for any renal or upper ureteral procedure.

The hidden incision endoscopic surgery (HIdES) port placement technique was developed to eliminate visible scarring (6). This involves placing the camera port and one robotic working port below the level of a Pfannenstiel incision and placing the second robotic working port infraumbilically (Figure 1B). Incisions are thus hidden beneath the underwear line, which has been shown to be preferable to patients and parents (6, 7).

After port placement, the next step is docking. The bed is rotated to raise the ipsilateral side, and the height of the bed is adjusted as desired. These changes must be made prior to robot docking unless using the Xi system with Trumpf Medical's TruSystem® 7000 dV OR table, which allows “integrated table motion” (OR table movement after docking). With the Si, docking is typically over the ispilateral shoulder at a slight angle or straight in from the side. Docking trajectory is more forgiving with the Xi system, as the robotic arms rotate on the boom into the optimal position when you perform anatomic targeting.

Next, the white line of Toldt is incised, and the colon is reflected medially to expose the retroperitoneum. One may alternatively utilize a transmesenteric approach for left-sided cases. The renal pelvis, UPJ, and ureter are then identified and dissected with limited, low-power cautery use. We routinely use a hitch stitch for traction to facilitate this dissection in the absence of an assistant port. We use a 4–0 Vicryl on an SH needle, which is manually straightened and passed directly through the abdominal wall by the assistant, through-and-through the renal pelvis, then back out the abdominal wall. The assistant may then adjust the tension as desired by the surgeon and snap the stitch in place at the level of the skin. A hitch stitch may not be necessary if the renal pelvis is not too floppy.

Dismemberment is the next step. Depending on UPJ configuration, one may choose an appropriate location and trajectory for renal pelvis transection in order to create an adequately wide pyelotomy for eventual anastomosis. If the UPJ insertion is high, an alternative is to ligate it, transect the proximal ureter, and create a new dependent pyelotomy for anastomosis. Non-dismembered techniques (e.g., Foley Y-V plasty or flap pyeloplasties) are preferred by some authors (8–10). Use of these methods has even been described in the setting of a crossing vessel, with concomitant cephalad translocation of the crossing vessel or Hellström technique (8, 9). Flaps can be particularly useful for long segments of UPJ/ureteral stricture, whereas a Heineke-Mikulicz type pelvotomy (Fenger-plasty) may be sufficient for short strictures (8, 11). We favor the classic Anderson-Hynes dismembered pyeloplasty technique in the majority of cases. Pelvic reduction may be performed if desired; however, this is rarely necessary in our experience.

The proximal ureter is then spatulated. Traditional descriptions favor spatulation along the lateral aspect because the proximal ureteral blood supply arises medially. Spatulation must be continued for an adequate length to ensure a wide anastomosis incorporating healthy ureter. A portion of the proximal ureter may ultimately be excised if it appears unsuitable for reconstruction; however, we recommend leaving such a segment attached for use as a handle until anastomosis is nearly complete. The anastomosis may be performed with running or interrupted fine absorbable suture. Typically, we perform half of the anastomosis with one running 5–0 Vicryl, then place a ureteral stent in antegrade fashion over a wire passed through an angiocatheter advanced directly through the abdominal wall. To confirm appropriate stent positioning, one may have the circulator instill dilute methylene blue solution into the bladder through the Foley catheter, which should reflux up through the stent if the distal coil is in the bladder. The proximal stent coil is then placed within the pelvis, and the anastomosis is completed with a second running suture. Alternative approaches include placing a stent in retrograde fashion or leaving a percutaneous nephrostomy/nephroureterostomy tube or Penrose drain instead of an internal stent. Tubeless procedures have also been described with no short-term complications (12). Long-term success rates of tubeless robotic pyeloplasty have yet to be determined.

Robotic pyeloplasty is effective, with multiple series including ≥50 patients reporting success rates of 94–100% utilizing a transperitoneal or retroperitoneal approach (2, 13–20). A recent retrospective long-term study reported an 8-year failure-free rate of 91.5% after robotic pyeloplasty (21). A meta-analysis from 2014 showed comparable success and complication rates in pediatric patients after minimally invasive or open pyeloplasty (22). A recent retrospective cohort study using the national Premier database revealed that while the total number of pyeloplasties decreased by 7% annually between 2003 and 2015, robotic cases increased by 29% annually, accounting for 40% of all cases in 2015 (23). Increased robot utilization was greatest in the pediatric population. Complication rates were similarly low in open and robotic cases.

### UPJ Reconstruction, Special/Complex Cases

Stones and/or UPJ polyps, if present, may be addressed concomitantly with retroperitoneoscopic or transperitoneal robotic pyeloplasty (24–27). Concurrent pyelolithotomy and pyeloplasty is safe and effective, with acceptable stone-free rates (94, 83, and 72% at 1, 3, and 6 months, respectively) (25). Operative time was longer for pyeloplasty with pyelolithotomy (median 151 min) vs. pyeloplasty alone (120 min, *p* < 0.0001), with no difference in length of hospital stay.

Ureteral fibroepithelial polyps are an uncommon but important source of obstructive hydronephrosis in children and can be challenging to diagnose preoperatively (24). If a polyp is suspected, endoscopy may be the preferred approach. However, in cases of large or multifocal lesions, or if a concurrent UPJ stenosis is thought to be present, robotics provide superior definitive management (26).

Redo (salvage) pyeloplasties present a special challenge. Dense peripelvic fibrosis, longer strictures, and compromised vascularity may all contribute to the increased difficulty in such cases. One recent study looking at laparoscopic redo pyeloplasties found that operative times were longer compared to primary cases (191 vs. 145 min, *p* = 0.0001), but success rates were comparable at 93.3% (28). Other groups have reported success rates from 77.8 to 100% for small cohorts undergoing redo pyeloplasty (29–32). Use of buccal mucosal onlay grafts for robotic salvage pyeloplasty (33, 34) and complex ureteral stricture repairs (35–37) has been shown to be safe and effective with short-term follow up.

Ureterocalicostomy is an option for renal salvage in cases where pyeloplasty is not feasible. The open procedure was originally described by Neuwirt (38). Indications for ureterocalicostomy are relative and may include UPJ obstruction in with an intrarenal pelvis or recurrent UPJ obstruction with dense scarring making redo pyeloplasty difficult or impossible. It has been considered a last resort for kidney preservation, as an alternative to nephrectomy (39). Robotic ureterocalicostomy was first reported in the pediatric population by Casale et al. with steps based on the open procedure (40). These authors retrospectively studied 9 pediatric patients who underwent transperitoneal robotic ureterocalicostomy in the setting of recurrent UPJ obstruction or intrarenal UPJ. Two patients underwent concomitant ureteroscopic stone treatment. The hilum was mobilized to allow for rapid vascular control; however, hilar clamping was not required in any case. Diuretic renogram confirmed unobstructed systems in all patients 12 months postoperatively (40).

## Mid Ureteral Reconstruction

### UU for mid Ureteral Stricture

RAL end-to-end UU may be indicated in the setting of mid ureteral stricture. Port placement for mid ureteral reconstruction can be achieved in a fashion similar to that described above for proximal ureteral reconstruction, with the ports shifted slightly inferiorly if needed. The diseased segment may be excised, and both ends spatulated at opposite aspects to achieve a wide anastomosis. For a relatively short stricture, a Heineke-Mikulicz repair may be adequate (41).

For long or multiple mid ureteral strictures, tension-free end-to-end anastomosis may not be possible. In such cases, the use of a graft may obviate the need for more morbid procedures such as ileal ureter, transureteroureterostomy, or autotransplantation. Buccal mucosal grafts may be used for complex pyeloplasties (33, 34) or complex ureteral stricture repairs (35–37). Use of the appendix as a ureteral substitute or as an onlay flap has also been described for complex right mid or upper ureteral stricture repair, initially in the open (42–44) or laparoscopic (45, 46) settings. Recently, Yarlagadda et al published a case report of robotic appendiceal interposition for right-sided ureteral stricture disease (47). In this case, a 5 cm obliterative ureteral stricture secondary to recurrent ureterolithiasis and pyelonephritis was repaired with interposition of the appendix between the proximal and distal healthy ureter. Resolution of hydronephrosis and flank pain was demonstrated at 10 months. Long-term results using this technique are needed.

## Lower Ureteral Reconstruction

### Extravesical Reimplantation for VUR

The most common RAL distal ureteral surgery is extravesical ureteral reimplantation for VUR, following steps of the open Lich-Gregoir technique originally described in the 1960s (48, 49) VUR may also be treated endoscopically or with open or laparoscopic transvesical reimplantation. Open ureteral reimplantation has a reported success of 93.5–98% (50–52). Endoscopic VUR treatment is the least invasive option, but is associated with variable radiographic cure rates of 67–93% (53–57). Success is likely dependent on technique, surgeon experience, and patient factors. The hydrodistention implantation technique (HIT) provides better outcomes than the older subureteric transurethral injection (STING) procedure, and several authors have reported radiographic success rates ≥80% (58–60). The Double HIT has emerged as the most common injection technique in the United States (61), affording the highest endoscopic success rates (57, 62).

Patient positioning for RAL distal ureteral procedures is typically lithotomy for the Si, allowing for cystoscopy (if desired) and robotic surgery in a single prep and drape. The robot is docked between the legs in this scenario. Side-docking is also possible, especially with the Xi, allowing the patient to remain supine. Port placement at our institution involves an 8.5 mm Si robotic camera port at the umbilicus with open Hasson or Veress technique. One may also use a 10 or 12 mm port (e.g., the Autosuture^®^ balloon trocar) for the Si robot (5). Xi camera and working ports are identical, allowing the camera to go through any of the ports.

After camera port placement, working ports are placed on either side of the umbilicus. These are placed inferiorly to the camera port to create a triangular working field for Si (Figure 1C), or in a line for the Xi. One may use 8 or 5 mm working ports for the Si, whereas only 8 mm instruments are available for the Xi. The HIdES port placement technique for lower urinary tract reconstruction involves placing the working ports at or below the level of a typical Pfannenstiel incision (Figure 1D) (6). Assistant port(s) and/or 3rd robotic port may be placed; however, we generally find this unnecessary. Unless the Xi and proprietary OR table are being used, one must adjust table height and position prior to docking.

**Figure 2:**
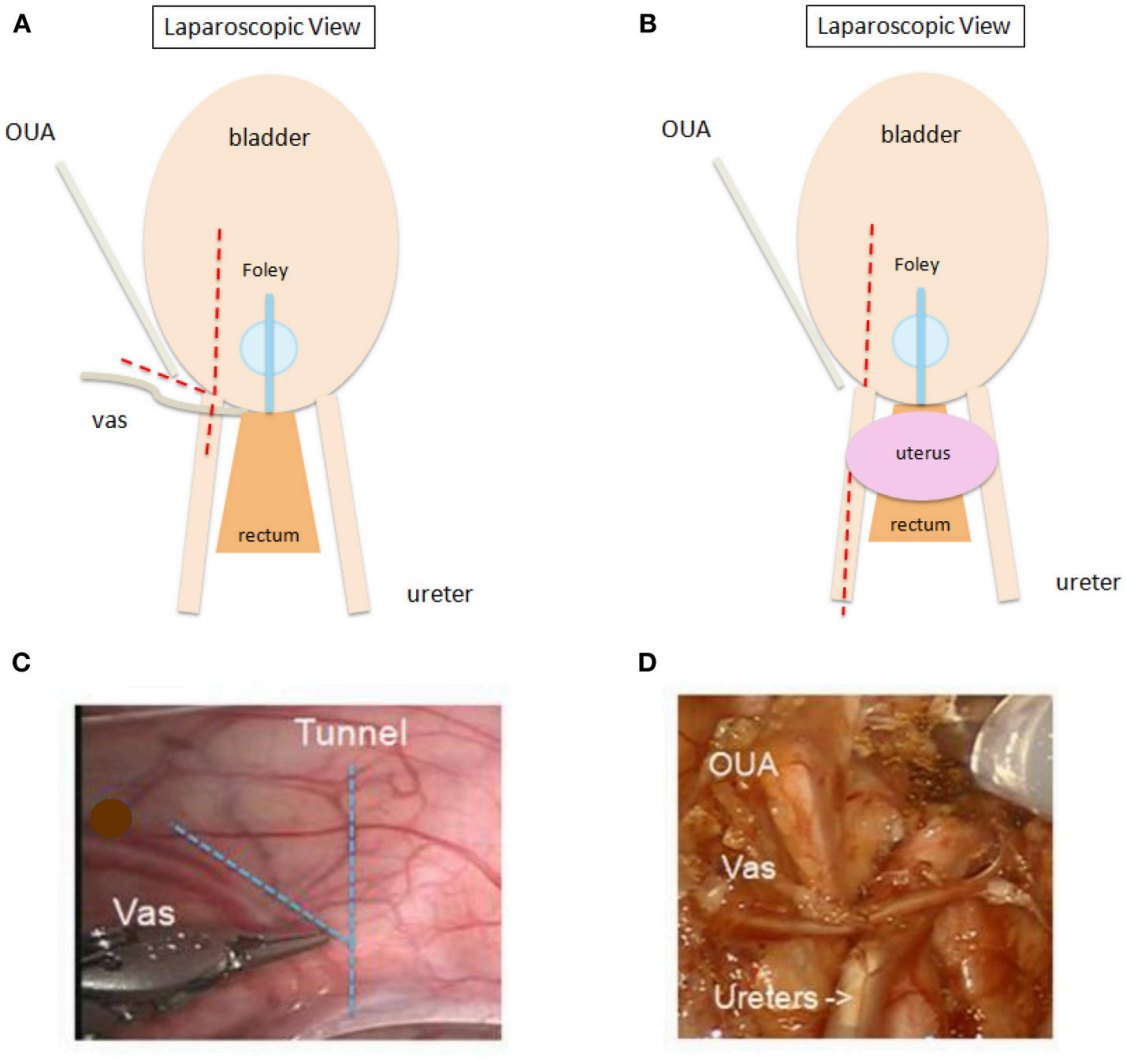
**(A,B)** Schematic showing sites for opening peritoneum (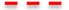) during RAL ureteral reimplantation. Peritoneum may be opened in line with proposed detrusor tunnel or transversely for wider exposure. “V” flap **(A)** recommended for adequately exposing vas deferens in boys. One may open peritoneum further cephalad along the ureter to allow for additional ureteral mobilization **(B)**, especially in peri- or post-pubertal girls or in otherwise complex cases. OUA, obliterated umbilical artery. **(C,D)** Intraoperative view prior to **(C)** and after **(D)** opening peritoneum in a male patient. OUA, obliterated umbilical artery.

Once docked, the first steps are opening the peritoneum (Figure 2) and mobilizing the ureter with judicious cautery use. The ureter is then followed distally to the ureterovesical junction (UVJ), taking care to preserve vas or uterine arteries in a boy or girl, respectively. A bladder hitch stitch may be utilized if the bladder is floppy and UVJ not clearly seen. A detrusor tunnel is created in the appropriate trajectory. The ideal location for detrusor tunnel may be more apparent in the absence of a hitch stitch, which may distort the anatomy. Ideal detrusor tunnel length has been described as 5:1 in comparison with the ureteral diameter (10). Flaps are developed on either side of the tunnel in order to prevent obstruction. Lastly, the tunnel is closed over the ureter. We use a running 3–0 V-loc for this, starting at the distal-most aspect and running proximally. Others may use different suture types, interrupted instead of running, and/or may start proximally, according to surgeon preference.

VUR resolution rates after extravesical RAL ureteral reimplantation (RALUR) reported in the literature range from 66.7 to 100% in multiple relatively small series (63–73). Overall success upon pooling these series is 91% (74). A multi-institutional retrospective study reported radiographic resolution in 87.9% of 280 ureters (75). More recently, a large prospective multi-institutional study reported 93.8% resolution in 199 ureters (76).

RALUR may be performed bilaterally; however, there is concern that bilateral dissection of the posterior bladder may disrupt the pelvic nerve plexus, resulting in higher rates of postoperative urinary retention. Nerve-sparing dissection has been proposed to reduce this complication (77). In 2008, Casale et al. reported a 97.6% success rate following bilateral nerve-sparing RALUR in 41 patients (65). There were no complications or instances of urinary retention. Herz et al. reported a 91.7% success rate for unilateral RALUR but a success rate of only 77.8% of ureters (72.2% of children) for bilateral cases (78). In this study, complication rates (including ureteral obstruction, readmission, and urinary retention) were higher for bilateral cases. A nerve-sparing technique was not utilized.

Peri-ureteral diverticula (if present) may be reduced/excised during reimplantation (79). In duplex systems, common sheath reimplantation with or without tapering has been described with good outcomes (80). Ureteral tapering may be performed while maintaining the native UVJ in the setting of a non-obstructed, refluxing megaureter (81). For complex reimplants (i.e., those with history of prior anti-reflux surgery, requiring tapering and/or dismembering, or associated duplication or diverticulum), Arlen et al. found comparable success and complication rates for RAL vs. open cases, with shorter hospitalization in the RAL group (82). Older children were more likely to undergo RALUR.

### Extravesical Reimplantation for UVJ Obstruction/Obstructed Megaureter

**Figure 3:**
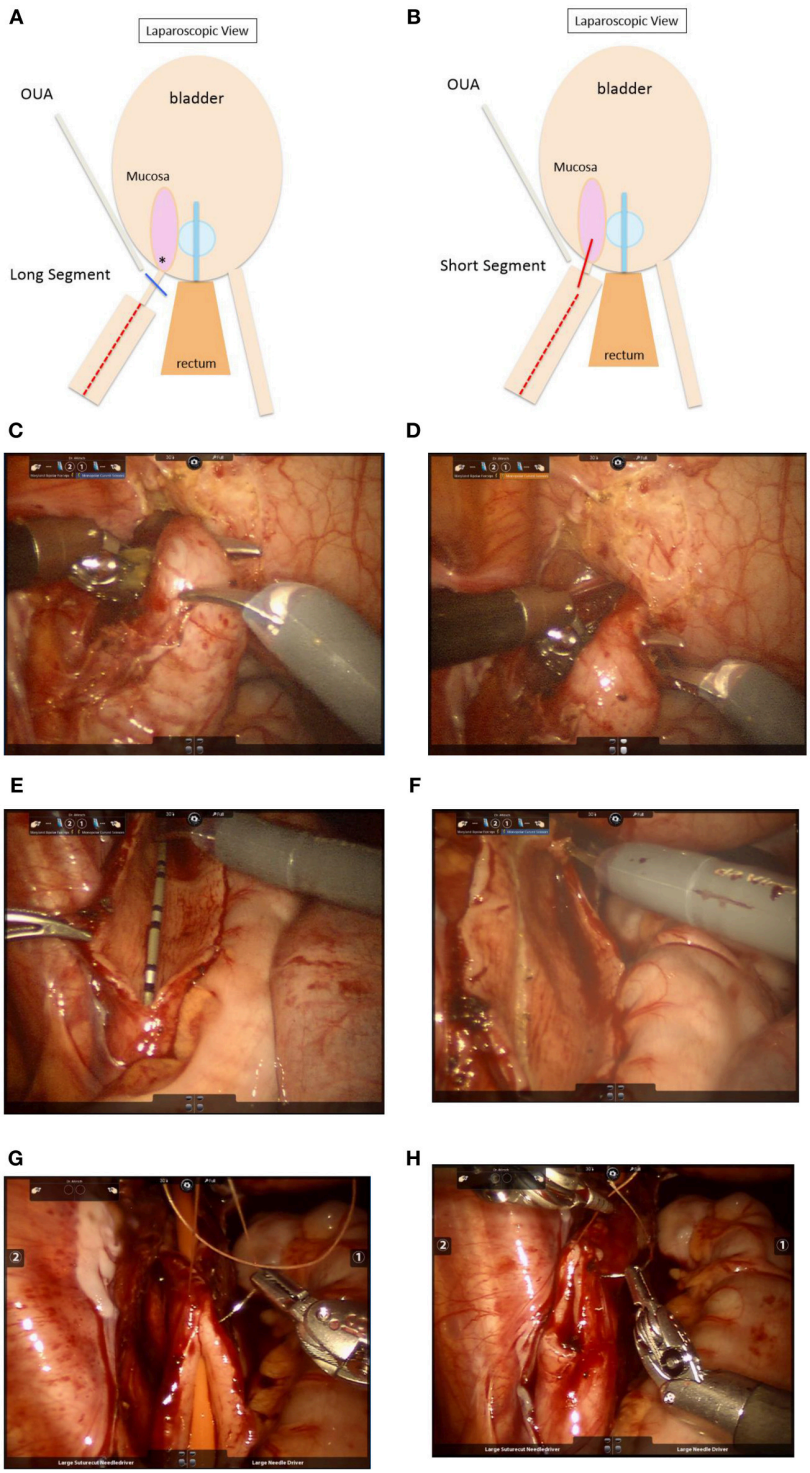
**(A)** Schematic showing repair of obstructed megaureter with a long segment of stenotic UVJ. Steps: i. Keep ureter attached. ii. Taper megaureter (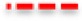) iii. Ligate UVJ (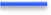). iv. Dismember ureter. v. Anastomosis at new site (*). Stent ± peritoneal closure. OUA, obliterated umbilical artery. **(B)** Repair of obstructed megaureter with a short stenotic UVJ segment. Steps: i. Keep ureter attached. ii. Taper megaureter (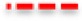) iii. Partially dismember (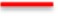). iv. *In situ* Heineke-Mikulicz anastomosis. Stent ± peritoneal closure. OUA, obliterated umbilical artery. **(C)** Intraoperative view during robotic repair of a left obstructed megaureter. The ureter has been mobilized circumferentially without devascularizing it. **(D)** The distally narrowed and obstructed segment is apparent in the view above. **(E)** A longitudinal ureterotomy has been created to allow for tapering. In this view, the ureter is still attached at the UVJ in order to maintain traction during tapering. **(F)** The ureter is scored to demarcate excess tissue for excisional tapering. **(G,H)** After excision of excess tissue, the ureter is closed/tapered using fine absorbable suture (5–0 Vicryl in the case above) over a 10 Fr catheter. The next steps include dismemberment at the UVJ, creation of ureteroneocystostomy, and creation of a detrusor tunnel to achieve a nonobstructed, nonrefluxing reimplantation.

RAL dismembered extravesical ureteral reimplantation with or without tapering may be used for repair of obstructed megaureters (Figures 3A,C–H) (83, 84). The obstructed UVJ is divided, and a new ureteroneocystostomy anastomosis is created. A non-refluxing detrusor tunnel is created as described above. When tapering, we prefer to leave the ureter connected to the bladder during this process to provide retraction. Dismemberment is then performed after tapering is complete, similar to the process described by Khan et al. (85). A non-dismembered technique may also be used to repair obstructed megaureters, using the Heineke-Mikulicz principle (Figure 3B) (86).

### UU in Duplex Systems

In appropriate duplex systems, end-to-side ureteroureterostomy (UU) can be performed proximally or distally depending on surgeon preference. We favor a distal approach, eliminating risk of hilar vessel injury, and allowing for intraoperative decision-making regarding performance of UU vs. ureteral reimplantation (vs. both concurrently in select settings). In some cases, it may be safer and more efficacious to perform UU in the mid ureter, thus avoiding both pelvic structures and renal hilar anatomy. Upper-to-lower UU may be performed for obstructed and/or ectopic upper moiety when there is no vesicoureteral reflux (VUR) into the lower moiety. Lower-to-upper UU may be performed in the setting of lower moiety VUR and unobstructed non-ectopic upper moiety (80).

Robot-assisted UU is a safe and effective alternative to open UU in children, with similar operative times and complication rates, and slightly shorter hospitalizations (87). UU has been shown to be safe and effective even in the setting of a minimally functioning/non-functioning moiety (as an alternative to upper moiety heminephrectomy) and irrespective of ureteral size difference (88).

When performing RAL UU, it is imperative to correctly identify each ureter. This can be facilitated with cystourethroscopy and passage of a temporary ureteral stent into one of the ureters. It is our practice to leave a double-J ureteral stent across the anastomosis, which is removed 4–6 weeks postoperatively. A renal-bladder ultrasound is performed ~4 weeks after stent removal, with additional imaging as clinically indicated.

## Conclusion

RAL surgery is a safe, minimally invasive technique with various applications in pediatric ureteric reconstruction. A robotic approach allows access to the ureter at all levels. Multiple aspects of robotic surgery, including magnified three-dimensional view and wristed movements with multiple degrees of freedom, are particularly well-suited to these delicate reconstructive procedures. Robotic surgery continues to enjoy growing popularity among urologists and patient families alike.

## Author Contributions

AB and AK both contributed to deciding the structure, content of the manuscript and to writing, editing the manuscript.

### Conflict of Interest Statement

The authors declare that the research was conducted in the absence of any commercial or financial relationships that could be construed as a potential conflict of interest.
